# Feasibility of paediatric schistosomiasis prevention with praziquantel, Madagascar

**DOI:** 10.2471/BLT.25.295521

**Published:** 2026-07-01

**Authors:** Valentina Marchese, Diavolana Koecher Andrianarimanana, Sonya Ratefiarisoa, Ariane Guth, Myriam Lassmann, Fiona Franz, Aaron Remkes, Jana Christina Hey, Lara Schramke, André Brito, Tahinamandranto Rasamoelina, Pia Rausche, Olivette Totofotsy, Alexina Olivasoa Zafinimampera, Tiana Randrianarisoa, Haingoniavo Garcia Rambeloson, Elveric Fesia Ratiaharison, Amadou Garba, Irina Kislaya, Clara Fabienne Rasoamanamihaja, Jürgen May, Rivo Andry Rakotoarivelo, Daniela Fusco

**Affiliations:** a Research Group: Implementation Research, Bernhard Nocht Institute for Tropical Medicine, Bernhard-Nocht-Strasse 74, Hamburg, D-20359, Germany.; b Centre Hospitalier Universitaire Androva, Mahajanga, Madagascar.; c German Center for Infection Research, Hamburg–Borstel–Lübeck–Riems, Germany.; d Centre d'Infectiolologie Charles Mérieux, Antananarivo, Madagascar.; e Association Promotion de la Santé, de l’Intelligence Artificielle et du Numérique, Fianarantsoa, Madagascar.; f Infectious Diseases Service, University Hospital Tambohobe, Fianarantsoa, Madagascar.; g Department of Control of Neglected Tropical Diseases, World Health Organization, Geneva, Switzerland.; h Department of Infectious Diseases Epidemiology, Bernhard Nocht Institute for Tropical Medicine, Hamburg, Germany.; i Ministry of Public Health, Government of Madagascar, Antananarivo, Madagascar.

## Abstract

**Objective:**

To assess the feasibility of fixed-dose, praziquantel preventive chemotherapy against schistosomiasis in young children in rural Madagascar, focusing on treatment uptake and sustainability.

**Methods:**

We used the Consolidated Framework for Implementation Research to guide the study. Between November 2022 and December 2023, children aged 9 to 24 months at various venues were offered 300 mg of praziquantel as a crushed half tablet mixed with water and sugar. We conducted a cross-sectional survey among caregivers to assess treatment uptake and acceptability, children’s compliance with drug administration, anxiety about adverse events, and praziquantel’s tolerability and safety. Factors associated with uptake and acceptability were identified using Poisson regression.

**Findings:**

The study analysis included 5142 child–caregiver pairs. The praziquantel uptake rate was 86.2% (95% confidence interval, CI: 85.3–87.1) and 80.2% (95% CI: 79.1–81.3) of caregivers regarded repeated preventive chemotherapy acceptable. Clear anxiety, and uncertainty, about adverse events were the most significant factors associated with refusal of both initial and repeated treatment. No children experienced serious adverse events or choking. Of the 4434 children who received praziquantel, 152 (3.4%) swallowed a partial dose, whereas 2467 (55.6%) cried and 1833 (41.3%) initially resisted treatment, but ultimately received it.

**Conclusion:**

Fixed-dose praziquantel preventive chemotherapy using a crushed half tablet with water and sugar was feasible in preschool-aged children and was potentially sustainable. This approach has a strong potential for scaling up in areas where schistosomiasis is highly endemic.

## Résumé

**Objectif:**

Évaluer la praticabilité d’une chimiothérapie préventive à dose fixe de praziquantel contre la bilharziose chez les jeunes enfants des zones rurales de Madagascar, en mettant l’accent sur l’administration du traitement et la pérennité de la mise en œuvre.

**Méthodes:**

Nous avons utilisé le cadre « Consolidated Framework for Implementation Research » pour orienter l’étude. Entre novembre 2022 et décembre 2023, des enfants âgés de 9 à 24 mois, dans différents lieux, se sont vus administrer 300 mg de praziquantel sous la forme d’un demi-comprimé écrasé mélangé à de l’eau et du sucre. Nous avons mené une étude transversale auprès de personnes s’occupant d’enfants afin d’évaluer l’administration du traitement et son acceptabilité, l’observance du traitement par les enfants, les craintes liées aux effets indésirables, ainsi que la tolérance et la sécurité du praziquantel. Les facteurs associés à l’administration du traitement et à son acceptabilité ont été identifiés à l’aide d’une régression de Poisson.

**Résultats:**

L’analyse de l’étude portait sur 5 142 couples enfant–personne en charge de l’enfant. Le taux d’administration du praziquantel était de 86,2% (intervalle de confiance (IC) à 95%: 85,3–87,1) et 80,2% (IC à 95%: 79,1–81,3) des personnes en charge considéraient comme acceptable une chimiothérapie préventive répétée. Des craintes manifestes et une incertitude quant aux effets indésirables constituaient les facteurs les plus importants associés au refus du traitement initial et des traitements répétés. Aucun enfant n’a présenté d’effets indésirables graves ni de risque d’étouffement. Sur les 4 434 enfants ayant reçu du praziquantel, 152 (3,4%) ont ingéré une dose partielle, tandis que 2 467 (55,6%) ont pleuré et 1 833 (41,3%) ont dans un premier temps manifesté une opposition au traitement mais l’ont finalement pris.

**Conclusion:**

La chimiothérapie préventive à dose fixe de praziquantel, consistant à administrer un demi-comprimé écrasé avec de l’eau et du sucre, s’est avérée praticable chez les enfants d’âge préscolaire et potentiellement pérenne. Cette approche présente un potentiel élevé de déploiement à grande échelle dans les zones où la bilharziose est hautement endémique.

## Resumen

**Objetivo:**

Evaluar la viabilidad de la quimioterapia preventiva con prazicuantel a dosis fija frente a la esquistosomiasis en niños pequeños de zonas rurales de Madagascar, centrándose en la aceptación del tratamiento y su sostenibilidad.

**Métodos:**

El estudio se guio por el Marco Consolidado para la Investigación sobre la Implementación. Entre noviembre de 2022 y diciembre de 2023, se ofrecieron 300 mg de prazicuantel a niños de entre 9 y 24 meses en distintos lugares de prestación de servicios, administrados como medio comprimido triturado y mezclado con agua y azúcar. Se realizó una encuesta transversal entre los cuidadores para evaluar la utilización del tratamiento, su aceptabilidad, la adherencia de los niños a la administración del medicamento, la preocupación por los acontecimientos adversos y la tolerabilidad y la seguridad del prazicuantel. Se identificaron mediante regresión de Poisson los factores asociados con la utilización del tratamiento y su aceptabilidad.

**Resultados:**

El análisis incluyó 5142 pares niño-cuidador. La tasa de utilización del prazicuantel fue del 86,2% (intervalo de confianza del 95%: 85,3-87,1), y el 80,2% de los cuidadores (IC del 95%: 79,1-81,3) consideró aceptable la administración repetida de quimioterapia preventiva. La preocupación y la incertidumbre acerca de los acontecimientos adversos fueron los factores más estrechamente asociados con el rechazo tanto del tratamiento inicial como del repetido. Ningún niño presentó acontecimientos adversos graves ni episodios de atragantamiento. De los 4434 niños que recibieron prazicuantel, 152 (3,4%) ingirieron una dosis parcial, mientras que 2467 (55,6%) lloraron y 1833 (41,3%) se resistieron inicialmente al tratamiento, aunque finalmente lo recibieron.

**Conclusión:**

La quimioterapia preventiva con prazicuantel a dosis fija, administrada como medio comprimido triturado con agua y azúcar, fue viable en niños en edad preescolar y presenta potencial de sostenibilidad. Este enfoque presenta un elevado potencial de ampliación en zonas donde la esquistosomiasis es altamente endémica.

## ملخص

**الغرض:**

تقييم جدوى العلاج الكيميائي الوقائي بجرعة ثابتة من برازيكوانتيل ضد داء البلهارسيا لدى الأطفال الصغار في المناطق الريفية بمدغشقر، مع التركيز على جرعة العلاج واستدامته.

**الطريقة:**

قمنا باستخدام الإطار العمل الموحد لأبحاث التنفيذ لتوجيه الدراسة. ما بين نوفمبر/تشرين ثاني 2022، وديسمبر/كانون أول 2023، تم إعطاء أطفال تتراوح أعمارهم بين 9 و24 شهرًا في مواقع مختلفة، 300 ملج من برازيكوانتيل على شكل نصف قرص مطحون ممزوج بالماء والسكر. قمنا بإجراء مسح قطعي بين مقدمي الرعاية لتقييم جرعة العلاج ومدى قبوله، والتزام الأطفال بتناول الدواء، والقلق بشأن الآثار الجانبية، ومدى تحمل برازيكوانتيل وسلامته. تم تحديد العوامل المرتبطة بجرعة العلاج وقبوله باستخدام نموذج التحوف لبواسون.

**الاستنتاج:**

شمل تحليل الدراسة 5142 زوجًا من الأطفال ومقدمي الرعاية. بلغ معدل جرعة البرازيكوانتيل %86.2 (بفاصل ثقة مقداره %95: 85.3 إلى 87.1)، و%80.2 (بفاصل ثقة مقداره %95: 79.1 إلى 81.3)، من مقدمي الرعاية اعتبروا العلاج الكيميائي الوقائي المتكرر مقبولاً. وكان القلق الواضح، وعدم اليقين بشأن الآثار الجانبية، من أهم العوامل المرتبطة برفض العلاج الأولي والمتكرر. لم يشهد الأطفال أية آثار جانبية أو حالات اختناق خطيرة. من بين 4434 طفلاً تلقوا البرازيكوانتيل، ابتلع 152 طفلاً (%3.4) العلاج بجرعات جزئية، بينما بكى 2467 طفلاً (%55.6)، وقاوم 1833 طفلاً (%41.3) العلاج في البداية، لكنهم تلقوه في النهاية.

**الاستنتاج:**

إن العلاج الكيميائي الوقائي ثابت الجرعة بالبرازيكوانتيل، باستخدام نصف قرص مطحون مع الماء والسكر، يُعدّ ممكناً للأطفال في سن ما قبل المدرسة، ومن المحتمل أن يكون مستداماً. يتمتع هذا الأسلوب بإمكانات قوية للتوسع في المناطق التي ينتشر فيها داء البلهارسيا بشكل مكثف.

## 摘要

**目的:**

评估固定剂量吡喹酮预防性化疗对马达加斯加农村地区低龄儿童血吸虫病的防治可行性，重点分析治疗覆盖率与长期可持续性。

**方法:**

我们采用实施研究整合框架指导本研究的开展。2022 年 11 月至 2023 年 12 月期间，我们在多个服务点为 9 至 24 月龄儿童提供 300 毫克吡喹酮，给药方式为将半片药片碾碎，兑糖水后服用。我们对儿童看护人开展了横断面调查，以评估治疗覆盖率和接受度、患儿的服药依从性、看护人对不良反应的担忧，以及吡喹酮的耐受性与用药安全性。我们采用泊松回归分析确定与覆盖率和接受度相关的因素。

**结果:**

本研究分析纳入 5142 对患儿与监护人作为研究对象。吡喹酮服用率为 86.2%（95% 置信区间 (CI)：85.3–87.1），并且 80.2%（95% CI：79.1–81.3）看护人可接受重复进行预防性化疗。对不良反应明显的担忧及不确定性是导致患儿拒绝接受首次和后续治疗的最主要影响因素。无患儿出现严重不良反应或噎呛。在 4434 名服用吡喹酮的儿童中，152 (3.4%) 名只咽下部分剂量的药物，2467 (55.6%) 名服药时哭闹；1833 (41.3%) 名起初抗拒服药，但最终均完成服药。

**结论:**

以将半片药片碾碎兑糖水服用的给药方式对学龄前儿童开展固定剂量吡喹酮预防性化疗是可行的，且具有长期可持续性。该方法具备在血吸虫病高流行地区大规模实施的巨大潜力。

## Резюме

**Цель:**

Оценить осуществимость химиотерапии фиксированной дозой празиквантела для профилактики шистосомоза у детей раннего возраста в сельских районах Мадагаскара, уделив особое внимание охвату лечением и устойчивости результатов.

**Методы:**

При проведении исследования мы руководствовались методологией Единой структуры исследований по внедрению (Consolidated Framework for Implementation Research). В период с ноября 2022 года по декабрь 2023 года детям в возрасте от 9 до 24 месяцев в различных учреждениях предлагали принять 300 мг празиквантела в виде измельченной половины таблетки с водой и сахаром. Авторы провели перекрестное исследование среди лиц, осуществляющих уход, для оценки охвата лечением и его приемлемости, соблюдения рекомендаций по приему препарата детьми, обеспокоенности по поводу нежелательных явлений, а также переносимости и безопасности празиквантела. Факторы, связанные с охватом и приемлемостью, были определены с помощью регрессии Пуассона.

**Результаты:**

В анализ исследования были включены 5142 пары «ребенок – лицо, осуществляющее уход». Уровень охвата лечением празиквантелом составил 86,2% (95%-й доверительный интервал, ДИ: 85,3–87,1), и 80,2% (95%-й ДИ: 79,1–81,3) лиц, осуществляющих уход, сочли повторную профилактическую химиотерапию приемлемой. Явная обеспокоенность и неуверенность по поводу нежелательных явлений были наиболее значимыми факторами, ассоциированными с отказом как от первоначального, так и от повторного лечения. Ни у одного ребенка не возникло серьезных нежелательных явлений или удушья. Из 4434 детей, принимавших празиквантел, 152 (3,4%) получили неполную дозу, тогда как 2467 детей (55,6%) плакали, а 1833 ребенка (41,3%) изначально сопротивлялись приему препарата, но в конечном итоге приняли его.

**Вывод:**

Профилактическая химиотерапия фиксированной дозой празиквантела с использованием измельченной половины таблетки с водой и сахаром оказалась осуществимой у детей дошкольного возраста и потенциально устойчивой. Этот подход имеет высокий потенциал для масштабирования в районах с высоким уровнем эндемичности шистосомоза.

## Introduction

Mass drug administration with praziquantel has long been the main public health strategy for combating schistosomiasis.^1^ Over time, the global approach has shifted from disease control to elimination,^2^ which has resulted in stronger recommendations on snail control, environmental management and access to safe water, and an expansion in the target group for mass drug administration from school-aged children to include adults and preschool-aged children, as outlined in the 2022 World Health Organization (WHO) guidelines.^3^ This shift was driven by growing evidence of schistosome infection and morbidity in preschool-aged children, the impact of community-wide treatment on transmission,^4^ and the increased availability of praziquantel.^5^ Nevertheless, operational challenges remain, particularly when including young children in praziquantel mass drug administration campaigns.^5^

Praziquantel is produced in 600 mg tablets, which can be halved for intake by adults and school-aged children, but only some brands allow accurate quartering for weight-based or height-based dosing for preschool-aged children.^6^ The drug’s bitter taste, even when crushed and mixed with sugar for young children, further reduces compliance.^5^^,^^7^ A paediatric formulation, orodispersible arpraziquantel, has been approved by the European Medicines Agency and partially addresses these issues.^8^ The initial roll-out of arpraziquantel started in some countries in 2025. However, supply is limited, only about 10 million tablets are available annually versus an estimated need for 50 million.^5^^,^^9^ Crushed praziquantel could provide an important complement to the paediatric formulation and increase coverage.

Another challenge is identifying effective delivery platforms. Experience with mass drug administration strategies for school-aged children and adults shows that coverage depends on the delivery method as well as on individual factors and that sustaining high coverage is difficult, especially for preschool-aged children.^10^^–^^12^ The integration of mass drug administration with existing child health programmes or community campaigns would be ideal but may face financial, logistic and sustainability constraints.^13^ WHO recommends three routine health visits for children aged 9 to 24 months.^14^ These visits provide opportunities for integrating schistosomiasis treatment into services at primary health-care centres and for ensuring that children receive at least one treatment before reaching school age.

In Madagascar, where schistosomiasis is prevalent and access to health care is limited, particularly in rural areas, school-based mass drug administration is the main treatment strategy.^15^ The aim of our study was to assess the feasibility of delivering fixed-dose praziquantel preventive chemotherapy to children aged 9 to 24 months in Madagascar through existing health systems and programmes.

## Methods

**Fig. 1:**
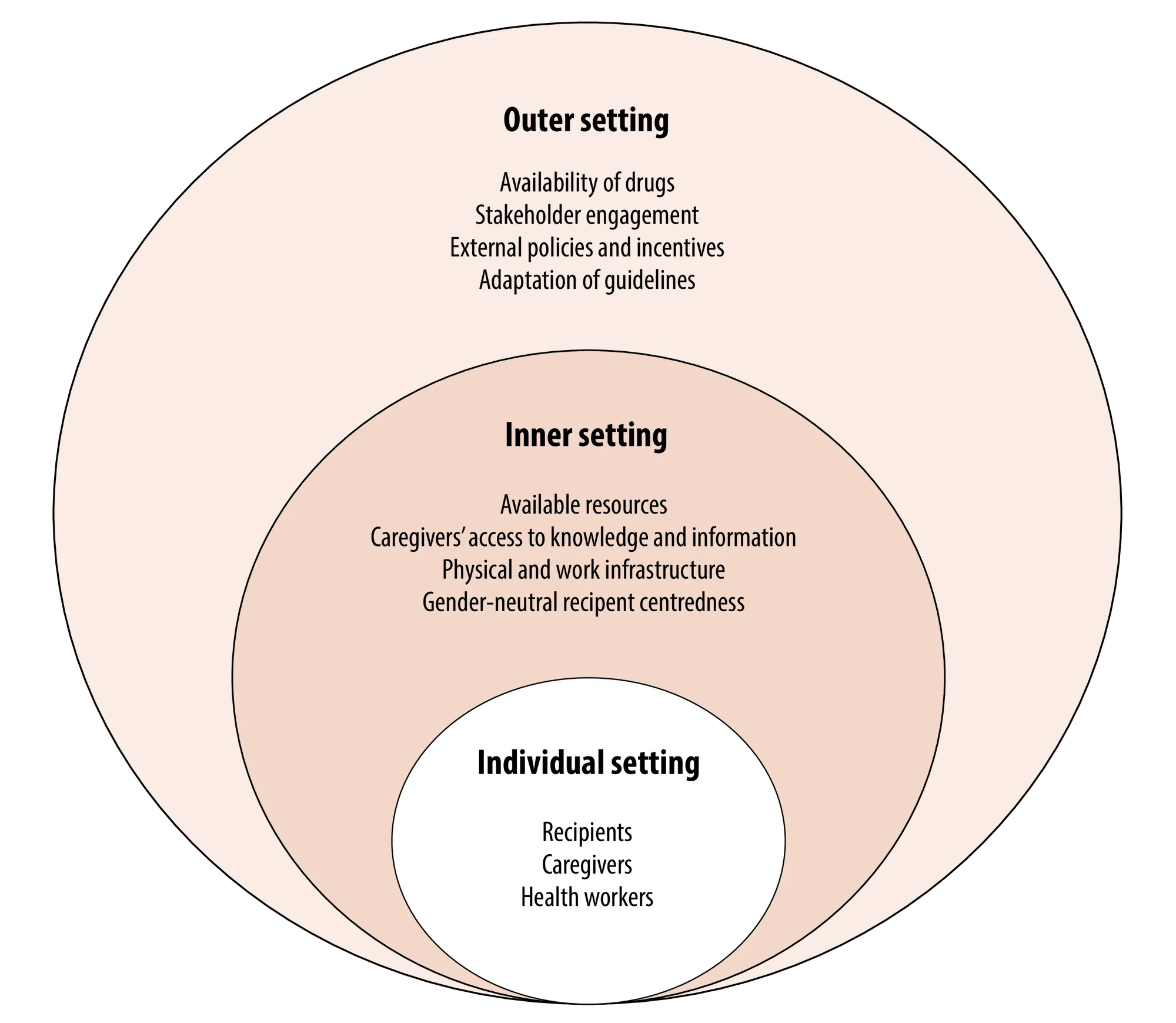
Adaptation of the Consolidated Framework for Implementation Research,^16^ study of praziquantel preventive chemotherapy for young children in rural Madagascar, 2023

Note: The availability of drugs refers to the local availability of praziquantel and drugs for the management of adverse events. See also Table 1.

We adapted the Consolidated Framework for Implementation Research to our study’s context by identifying specific framework subdomains and process steps that could influence both the implementation and outcomes of praziquantel preventive chemotherapy for young children (Fig. 1).^16^ Our intervention was designed in collaboration with Madagascar’s National Schistosomiasis Control Programme, local academic stakeholders and the national reference centre for infectious disease surveillance, the *Centre d'Infectiologie Charles Mérieux.* We identified critical elements to be added to the Framework (Table 1) through a collaborative rapid assessment approach that involved meetings with our partners and interviews with key informants, such as community leaders, facility managers and national stakeholders.^17^ Ethical approval was obtained from the National Ethics Committee of Madagascar (N°019MSANP/SG/AMM/CERBM) and the Hamburg State Medical Chamber’s Ethics Committee in Germany (2022–100935-BO-ff).

**Table 1:** Critical subdomains of the Consolidated Framework for Implementation Research,^16^ implementation of praziquantel preventive chemotherapy for young children in rural Madagascar, 2023

Framework domain, critical subdomain	Action
**Outer setting**	
Drug availability	Establish a supply chain for praziquantel procurement and identify medical coordinators
Stakeholder engagement	Engage health workers in the local health system
External policies and incentives	Non-modifiable within the scope of the project
Adapted guidelines	(i) Develop standard operating procedures that cover training for drug administration and the management of adverse events; (ii) reinforce adverse event surveillance through linkage to Madagascar’s national surveillance system for adverse events; and (iii) strengthen capacity for adverse event management
**Inner setting**	
Resources available	Identify local resources, including equipment at primary health-care centres, supplies of consumables (e.g. drugs to treat adverse events, such as corticosteroids, paracetamol, antihistamines and antidiarrheals) and medical supplies
Caregivers’ access to knowledge and information	Integrate items on knowledge and information into the survey questionnaire to inform awareness campaigns tailored to the local context
Physical and work infrastructure	Coordinate with local authorities to secure suitable spaces in health-care facilities for the intervention
Gender-neutral recipient-centredness	Integrate items on the importance of gender into the survey questionnaire to inform awareness campaigns tailored to the local context
**Individual setting**	
Recipient’s compliance with praziquantel administration	Include dedicated items on choking-related adverse events and children’s compliance with treatment in the survey questionnaire
Caregivers (i.e. key decision-makers)	Identify factors associated with praziquantel uptake and the acceptability of repeated preventive chemotherapy (i.e. individual routinization)
Health workers (i.e. key influencers)	Arrange training and information sessions for local health workers

### Planning and implementation

All partners met weekly during the 12 weeks before study initiation (i.e. from November 2022 to January 2023) and throughout the intervention. We collected qualitative data through semi-structured interviews with community health workers, community members and national stakeholders using open-ended questions on the principal topics of interest, such as drug availability, site accessibility and perceived environmental safety. Study sites were specially selected to assess the feasibility of the intervention, the integration of the intervention at different health-care levels, and variations in caregiver behaviour outside of health-care settings. Before implementation, the primary health-care centre infrastructure and drug availability at each site were assessed and supplies for the management of adverse events and praziquantel supplies were secured. Two medical coordinators managed the supply chain, supervised study implementation, data collection and adverse event management, and reported to the national surveillance system for adverse events. In addition, 22 health workers underwent one day’s training in basic adverse event management, which was further addressed in three meetings with the head doctors at primary health-care centres. A patient transfer protocol using private transport was established for cases requiring inpatient care and standard operating procedures were developed to support the intervention. Weekly reports on study enrolment, praziquantel administration, adverse events and consumable use were produced, which enabled local stakeholders to make responsive adjustments.

The intervention lasted 14 months and took place in the Boeny region of north-western Madagascar, where *Schistosoma haematobium* is highly endemic (i.e. endemicity greater than 50%) and where there are health system and sociodemographic fragilities:^15^ most residents live in rural areas, 6.5% of the population is younger than 5 years, and access to schools, health care, clean water and sanitation remains limited.^18^ The intervention involved 13 primary health-care centres, one district hospital and two community sites, which were selected on the recommendations of key informants and on the basis of local epidemiological and demographic data. Caregiver–child pairs were recruited in four contexts: (i) at primary health-care centres during unplanned consultations; (ii) at primary health-care centres during routine paediatric or immunization visits; (iii) at the district hospital during unplanned consultations; and (iv) in places where the community gathered.

For inclusion in the study, caregivers had to give written informed consent in Malagasy and children had to be aged between 9 and 24 months. Exclusion criteria included ongoing pathological conditions, epilepsy, antiepileptic or antitubercular treatment with rifampicin or rifapentine, and a known allergy to praziquantel.

Children received a fixed 300-mg dose of praziquantel after a snack (i.e. a biscuit and fruit juice) in the form of a crushed half 600-mg tablet mixed with water and sugar. This dose was based on WHO’s recommended dose range of 30 to 60 mg/kg adapted to Malagasy weight-for-age and height-for-age estimates.^19^^,^^20^ Mean weight-for-age and height-for-age values for Malagasy children aged 9 to 24 months were reported to fall between the −1 and −2 *z*-scores for WHO child growth standards,^21^ which meant the dose administered was consistent with praziquantel dosage recommendations given by the height-based extended dose pole.^22^^,^^23^ Praziquantel administration was observed by health workers, who used comforting strategies such as encouraging small sips and pauses to ease ingestion. If the child spat out a substantial amount, no additional dose was given for safety reasons and treatment was considered inadequate.

### Intervention assessment

We assessed two main outcomes of the intervention: (i) treatment uptake by children; and (ii) the sustainability of the intervention among caregivers, where we defined sustainability as the proportion of caregivers who regarded repeated preventive chemotherapy for children as acceptable.In addition, the safety and tolerability of treatment, treatment compliance and satisfaction with treatment and health workers were also evaluated **(**Table 2).^24^^–^^26^ Outcomes were measured using a cross-sectional study design with data collected between February and December 2023.

**Table 2:** Study outcomes, implementation of praziquantel preventive chemotherapy for young children in rural Madagascar, 2023

Outcome	Definition	Data source	Data analysis
**Primary outcome**			
Treatment uptake	Ratio of the number of children treated with praziquantel (with their caregivers’ consent) to the number of children enrolled in the study	Answer to the survey questionnaire item: “If we offer free schistosomiasis treatment to children aged 9 to 24 months, would you now agree to your child being treated?” (Response options: yes; no; and maybe but not today)	(i) Responses were combined into two dichotomous outcomes: no and maybe but not today versus yes; and (ii) Poisson regression was used to identify factors associated with treatment uptake
Sustainability of the intervention	Percentage of caregivers who regarded repeated preventive chemotherapy for children as acceptable	Answer to the survey questionnaire item: “Do you think it would be a good idea to offer praziquantel for the treatment of schistosomiasis as a regular treatment for children?” (Response options: yes; no; and I do not know)	(i) Responses were combined into two dichotomous outcomes: yes versus no and I do not know; and (ii) Poisson regression was used to identify factors associated with acceptance of repeated preventive chemotherapy
**Secondary outcome**			
Treatment compliance	Health workers’ observation of a child resisting treatment, crying or having an adverse swallowing event (e.g. choking, spitting or vomiting) during drug administration^24^	Treatment compliance events were reported systematically by health workers and through the survey questionnaire	Descriptive analysis
Treatment tolerability	The appearance of adverse events in the 30 minutes after praziquantel administration	(i) Health workers systematically reported vomiting, rashes, oedema, fever and inconsolable crying; (ii) any additional sign or symptom was reported in response to open-ended questions on the survey questionnaire; and (iii) side-effects were graded for severity	Descriptive analysis
Treatment safety	The occurrence within 30 days of receiving praziquantel of death, hospitalization or any unusual or rare adverse event classified by health workers as grade 3 to 5 in severity^a^	(i) Active surveillance by health workers in the 30 minutes after praziquantel administration; and (ii) passive surveillance by caregivers who reported to study health workers until 30 days after administration	Descriptive analysis
Sustainability (i.e. continued delivery of the intervention)	(i) Number of trained personnel available; (ii) adequate provision of drugs to primary health-care centres; (iii) development of standard operating procedures; and (iv) integration of praziquantel preventive chemotherapy into the health-care system	(i) Weekly meetings with study partners; (ii) interviews with key informants, such as community leaders, facility managers and national stakeholders; and (iii) weekly study reports	(i) Collaborative, rapid assessments; and (ii) reviews of project reports

^a^ Any grade 3 to 5 adverse event has to be reported to Madagascar’s Ministry of Health in accordance with national pharmacovigilance procedures.

### Data management

Trained health workers conducted face-to-face interviews with caregivers in French or Malagasy using a structured, paper-based questionnaire. The questionnaire had five sections: (i) the sociodemographic characteristics of the child and caregiver; (ii) the caregiver’s awareness and knowledge of schistosomiasis and praziquantel; (iii) the caregiver’s willingness to accept, and perception of, praziquantel treatment; (iv) the caregiver’s satisfaction with treatment and health workers; and (v) the child’s compliance with treatment, and adverse events.

Data were checked manually for missing entries by health workers involved in the study and medical coordinators, and data were entered into a REDCap database (Vanderbilt University, Nashville, United States of America) using double data entry.^27^ Quality control of data processing and validation was undertaken at regular intervals during and at the end of data entry. An external data manager monitored the data entered and requested and recorded checks and amendments.

### Sample size

Based on operational considerations, such as the availability of resources and an expected treatment capacity of a maximum of 10 individuals per day, the total number of caregiver–child pairs to be recruited was set at 5200. This sample size was sufficient to detect a 2 percentage point difference in praziquantel uptake from the WHO threshold for successful mass drug administration of 75%, in either direction, at a 5% significance level and with a power of 90%. The sample size also enabled meaningful evaluation of the sustainability of the intervention, the tolerability of praziquantel, and adverse events. A convenience, non-probability sampling strategy was employed to select caregiver–child pairs.

### Statistical analysis

The characteristics of the children and caregivers are reported using descriptive statistics and include both absolute and relative frequencies. The proportions of caregivers who agreed to praziquantel treatment, who refused praziquantel and who regarded repeated preventive chemotherapy as acceptable were estimated with 95% confidence intervals (CIs). To identify factors associated with treatment refusal and the acceptance of repeated preventive chemotherapy, we estimated crude and adjusted prevalence ratios (PRs) using Poisson regression models with robust standard errors. Separate models were fitted for each outcome. Median satisfaction scores were estimated for caregivers' satisfaction with the treatment offered and with health workers, and the significance of the difference between caregivers who accepted praziquantel treatment and those who refused it was estimated using the Mann–Whitney *U* test. Adverse events among praziquantel-treated children are reported using absolute and relative frequencies. Observations that included missing data on outcomes or independent variables were excluded from the analysis. A two-sided *P*-value of less than  0.05 was considered significant.

## Results

### Conceptualization of the intervention

Elements in the outer setting (Fig. 1) that emerged as barriers to the treatment of young children included: (i) local availability of praziquantel and drugs for the management of adverse events; (ii) stakeholder engagement; (iii) external policies and incentives; and (iv) the need for adapted guidelines. In the inner setting, we identified the following factors as critical for effective implementation of the treatment strategy: (i) the resources available; (ii) the physical and work infrastructure available for treatment; and (iii) culture (e.g. caregivers’ recipient-centredness and knowledge). Finally, both the users (i.e. children and caregivers) and providers of the health service were identified as critical to the success of the intervention (Fig. 1). Following consolidation meetings with stakeholders, the intervention was conceptualized and approved by all involved.

### Treatment uptake

In total, 5163 caregiver–child pairs were recruited. Following data quality checks, 5142 pairs were included in the analysis (details are available in the online repository).^28^ Most participants (77.2%; 3971/5142) were recruited at primary health-care centres during unplanned consultations, followed by 16.9% (868/5142) recruited at primary health-care centres during routine paediatric or immunization visits (Table 3). In addition, 4.0% (207/5142) and 1.9% (96/5142) were recruited at the district hospital and at community gathering places, respectively. The sociodemographic characteristics of caregivers and children are also reported in Table 3. Praziquantel uptake was accepted by 86.2% (4434/5142; 95% CI: 85.3–87.1) of caregivers, whereas 13.8% (708/5142; 95% CI: 12.9–14.7) refused treatment.

**Table 3:** Characteristics of caregivers and children, study of praziquantel preventive chemotherapy for young children in rural Madagascar, 2023

Characteristic	No. (%) (*n* = 5 142)
**Recruitment context**	
Primary health-care centre during an unplanned consultation	3971 (77.2)
Primary health-care centre during a routine paediatric or immunization visit	868 (16.9)
District hospital during an unplanned consultation	207 (4.0)
Community gatherings	96 (1.9)
**Child’s sex^b^**	
Female	2579 (50.2)
Male	2563 (49.8)
**Child’s age, in months**	
9–12	1699 (33.0)
13–17	1590 (30.9)
18–24	1853 (36.0)
**Caregiver had health insurance**	
No	4141 (80.5)
Yes	1001 (19.5)
**Caregiver’s type**	
Mother	4662 (90.7)
Father	254 (4.9)
Other^a^	226 (4.4)
**Caregiver’s sex^b^**	
Female	4864 (94.6)
Male	278 (5.4)
**Caregiver’s age, in years**	
< 25	1999 (38.9)
≥ 25 to 34	2375 (46.2)
≥ 35	768 (14.9)
**Caregiver’s educational level**	
No education	410 (8.0)
Primary	1979 (38.5)
Secondary	2361 (45.9)
Tertiary	392 (7.6)
**Caregiver’s marital status**	
Married	4376 (85.1)
Unmarried	766 (14.9)
**Caregiver’s occupation**	
Farmer	3177 (61.8)
Non-farmer	1965 (38.2)
**Caregiver accompanied by another person**	
No	3608 (70.2)
Yes	1534 (29.8)

^a^ The other caregivers category included extended family members: there were 104 grandparents, 100 aunts, 13 uncles, 7 sisters, 1 brother and 1 cousin.

^b^ No other sex was reported.

Note: inconsistencies may arise in some values due to rounding.

Of the 5142 caregivers, 4202 (81.7%) considered children to be at risk of schistosome infection and 2653 (51.6%) believed the infection could present asymptomatically (Table 4). Moreover, 2011 (39.1%) reported prior family experience with praziquantel preventive chemotherapy, whereas 2123 (41.3%) reported no experience. Praziquantel was generally perceived positively: 65.8% ( 3385/5142) of respondents expressed favourable views regarding its perceived efficacy against schistosomiasis and only 15.2% (784/5142) reported anxiety about adverse events.

**Table 4:** Caregivers' knowledge and perceptions of schistosomiasis and praziquantel, study of praziquantel preventive chemotherapy for young children in rural Madagascar, 2023

Caregivers’ knowledge or perception	No. (%) (*n* = 5 142)
**Previous family experience with praziquantel preventive chemotherapy**	
No	2123 (41.3)
Yes	2011 (39.1)
I do not know	1008 (19.6)
**Accepted repeated praziquantel preventive chemotherapy for children**	
No	155 (3.0)
Yes	4124 (80.2)
I do not know	863 (16.8)
**Anxiety about adverse events**	
No	3103 (60.4)
Yes	784 (15.2)
I do not know	1255 (24.4)
**Perception that praziquantel is effective against schistosomiasis**	
No	317 (6.2)
Yes	3385 (65.8)
I do not know	1440 (28.0)
**Knowledge of schistosomiasis transmission routes**	
No	965 (18.8)
Yes	4177 (81.2)
**Aware of children’s risk of schistosome infection**	
No	174 (3.4)
Yes	4202 (81.7)
I do not know	766 (14.9)
**Aware that schistosomiasis can be asymptomatic in children**	
No	790 (15.4)
Yes	2653 (51.6)
I do not know	1699 (33.0)
**Aware of children’s risk of reinfection after praziquantel treatment**	
No	1206 (23.5)
Yes	2712 (52.7)
I do not know	1224 (23.8)

Note: inconsistencies may arise in some values due to rounding.

### Factors influencing uptake

**Fig. 2:**
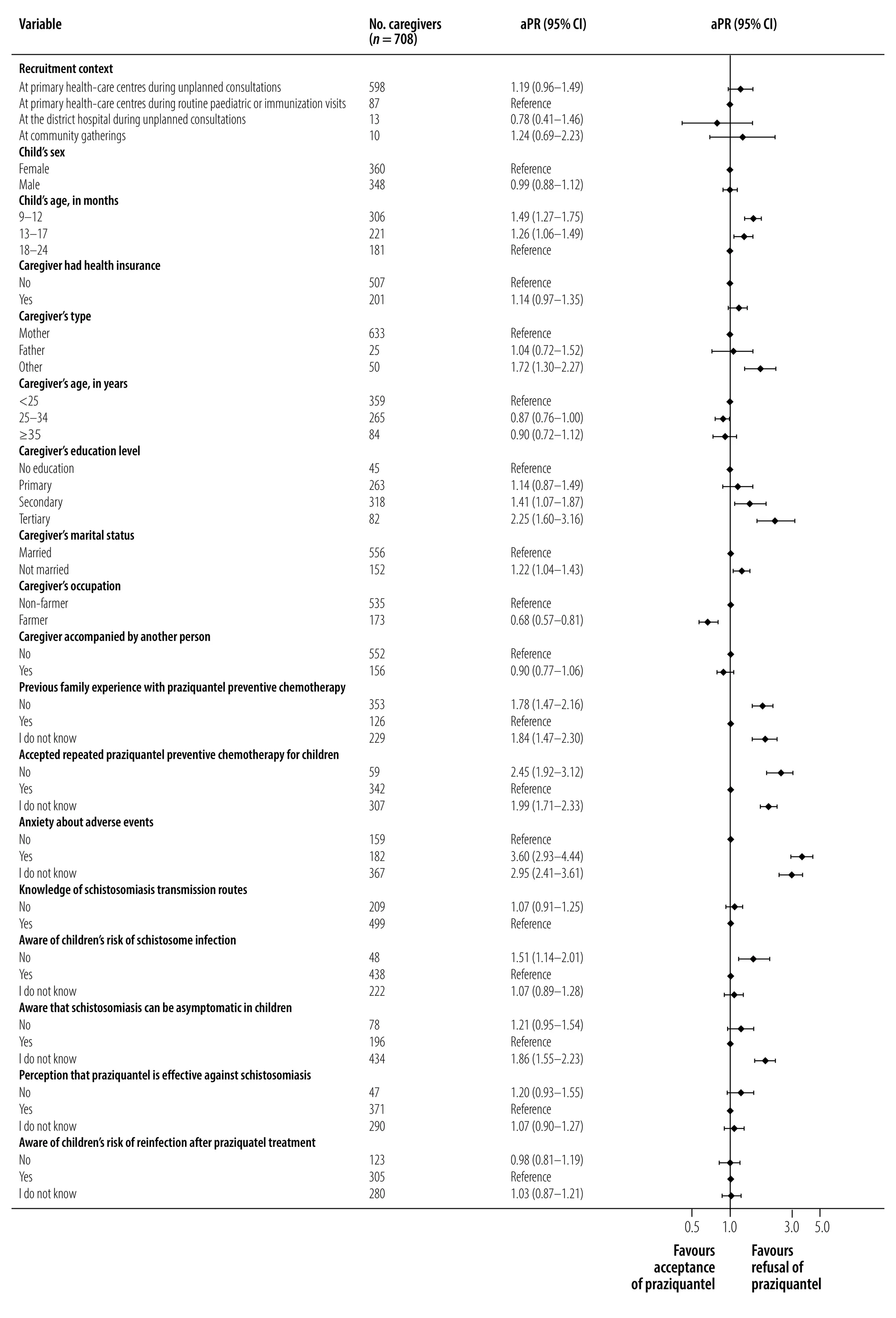
Factors associated with caregivers’ refusal of praziquantel preventive chemotherapy for their child in rural Madagascar, 2023

Overall, 708 caregivers refused praziquantel treatment for their child. Refusal was significantly associated with: (i) secondary (adjusted PR, aPR: 1.41; 95% CI: 1.07–1.87) and tertiary (aPR: 2.25; 95% CI: 1.60–3.16) education, relative to no education; (ii) not being the child’s parent, relative to being the child’s mother (aPR: 1.72; 95% CI: 1.30–2.27); and (iii) being unmarried, relative to being married (aPR: 1.22; 95% CI: 1.04–1.43). Crude PRs are reported in the online repository.^28^ In contrast, being a farmer was inversely associated with the refusal of praziquantel (aPR: 0.68; 95% CI: 0.57–0.81). No significant association was observed with the child’s sex. However, refusal was inversely associated with the child’s age: relative to the caregivers of children aged 18 to 24 months, the aPR for refusal was 1.49 (95% CI: 1.27–1.75) for the caregivers of children aged 9 to 12 months and 1.26 (95% CI: 1.06–1.49) for the caregivers of children aged 13 to 17 months. A lack of awareness of children’s risk of schistosomiasis was also associated with refusal (aPR: 1.51; 95% CI: 1.14–2.01), as was no, or uncertainty about, previous family experience with praziquantel preventive chemotherapy versus previous experience: the aPR was 1.78 (95% CI: 1.47–2.16) and 1.84 (95% CI: 1.47–2.30), respectively. Anxiety about adverse events showed the strongest association with refusal: the aPR was 3.60 (95% CI: 2.93–4.44) relative to caregivers with no anxiety and that association remained significant even among caregivers who were uncertain about adverse events (aPR: 2.95; 95% CI: 2.41–3.61; Fig. 2).

aPR: adjusted prevalence ratio; CI: confidence interval.

Note: All children were classified as male or female.

### Sustainability of praziquantel treatment

The praziquantel intervention continued consistently for 44 weeks, with an average of 117 caregiver–child pairs included per week. Regular feedback on implementation challenges was collected by the study team. The sustainability assessment found that 80.2% (95% CI: 79.1–81.3) of caregivers (4124/5142) regarded repeated preventive chemotherapy as acceptable (Table 4;.

**Fig. 3:**
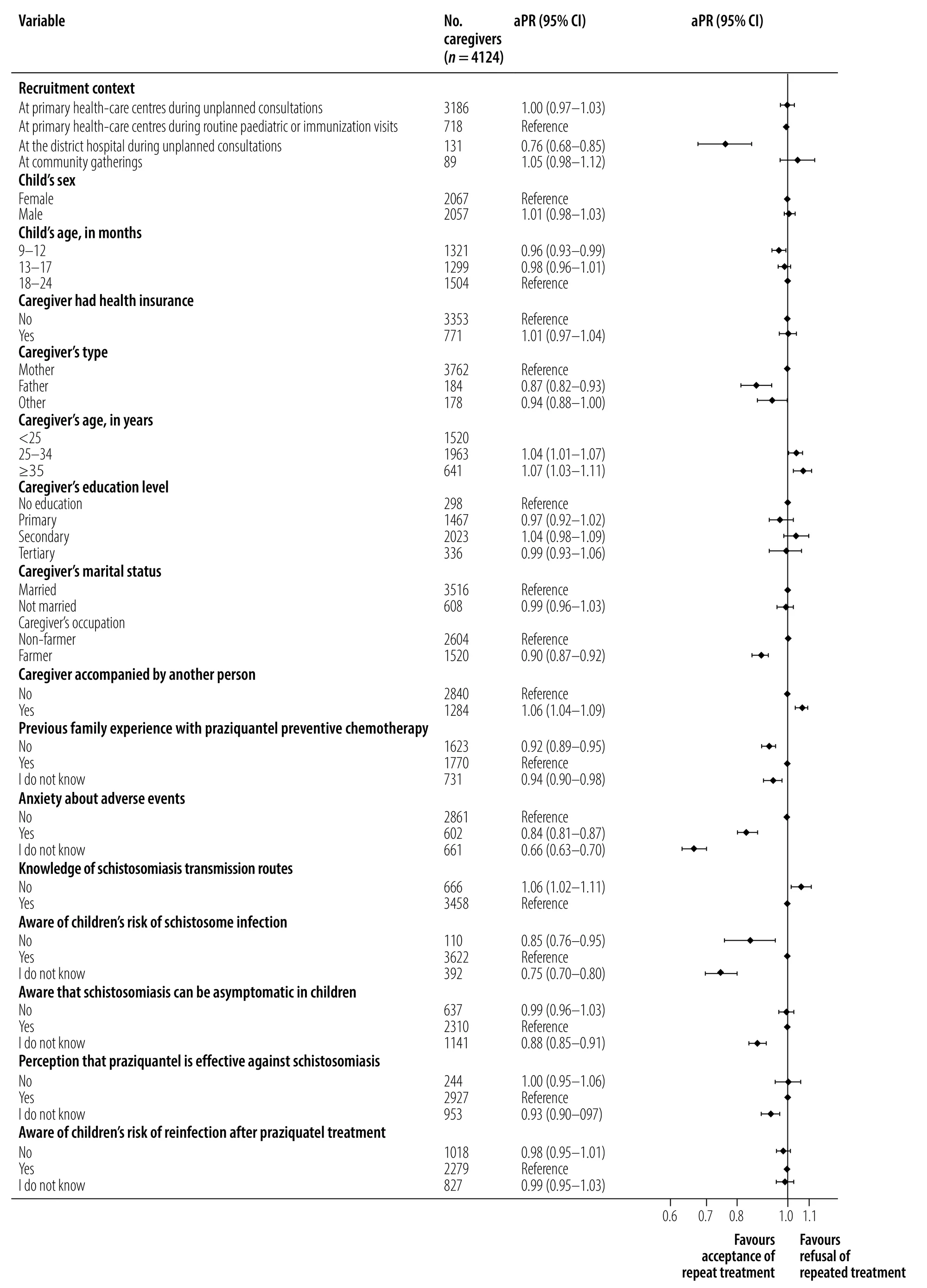
Factors associated with acceptance of repeated treatment, praziquantel preventive chemotherapy for young children in rural Madagascar, 2023

There was a significant positive association between acceptance of repeated treatment and the caregiver’s age (Fig. 3): relative to those younger than 25 years, the aPR for acceptance was 1.04 (95% CI: 1.01–1.07) for those aged 25 to 34 years and 1.07 (95% CI: 1.03–1.11) for those aged over 34 years. However, relative to being a mother, there were inverse associations with being a father (aPR: 0.87; 95% CI: 0.82–0.93) and being a caregiver who was not the biological mother (aPR: 0.94; 95% CI: 0.88–1.00). Compared with not being a farmer, being one was also inversely associated with acceptance of repeated treatment (aPR: 0.90; 95% CI: 0.87–0.92). In addition, relative to caregivers recruited during routine paediatric or immunization visits, those recruited at the district hospital were less likely to favour repeated treatment (aPR: 0.76; 95% CI: 0.68–0.85), whereas there was no significant association with recruitment in any other context. Uncertainty about, or a lack of awareness of, the risk of schistosome infection, the risk of reinfection after praziquantel treatment or the possibility of asymptomatic infection among children and no, or uncertainty about, previous family experience with praziquantel preventive chemotherapy were all associated with a reluctance to accept repeated treatment. However, the strongest negative association with acceptance was anxiety about adverse events: relative to those who were not anxious, the aPR for acceptance was 0.84 (95% CI: 0.81–0.87) for caregivers who were clearly anxious and 0.66 (95% CI: 0.63–0.70) for those who reported being unsure whether they were anxious. Crude PRs are reported in the online repository.^28^

aPR: adjusted prevalence ratio; CI: confidence interval.

Note: All children were classified as male or female.

### Treatment compliance, adverse events and service satisfaction

Among the 4434 children treated with praziquantel, 2467 (55.6%; 95% CI: 54.2–57.1) cried and 1833 (41.3%; 95% CI: 39.9–42.8) initially resisted but ultimately received treatment. Only 152 (3.4%; 95% CI: 2.9–4.1) spat during praziquantel administration. There were no episodes of choking. Fifty-two children experienced adverse events within 30 minutes after treatment (1.2%; 95% CI: 0.9–1.5): the most common were vomiting, in 29 (0.7%; 95% CI: 0.5–0.9), and drowsiness, in 22 (0.5%; 95% CI: 0.3–0.8). Details are available in the online repository.^28^ No deaths or serious adverse events were reported within 30 days of treatment.

Caregivers who accepted praziquantel treatment reported significantly higher satisfaction with study health workers than those who refused: the median satisfaction score was 9 (interquartile range, IQR: 8–10) and 8 (IQR: 7–9) in the two groups, respectively (*P*-value: < 0.001); corresponding median satisfaction scores in the two groups for the treatment offered were 9 (IQR: 8–10) and 8 (IQR: 7–9), respectively (*P*-value: < 0.001). Details are available in the online repository.^28^

## Discussion

Our study demonstrated that preventive chemotherapy for schistosomiasis using crushed, fixed-dose praziquantel tablets in children aged 9 to 24 months was feasible in rural Madagascar. Most children received praziquantel, and treatment was highly tolerable and compliance was good. In addition, most caregivers regarded repeated praziquantel treatment for their child as acceptable. Moreover, our approach proved child-friendly; fewer than 5% of children were inadequately treated. The main factor associated with praziquantel refusal was caregiver anxiety about adverse events.

Use of 300-mg half tablets simplified treatment delivery while meeting WHO’s recommended dose range for Malagasy children, based on weight-for-age and height-for-age estimates.^18^ Although there are concerns about undertreatment and resistance, the low adverse event rate of 1.2% we observed and evidence from higher-dose studies suggest that the use of entire 600-mg tablets could further simplify administration.^29^

This approach is probably applicable to similar settings. However, factors influencing praziquantel uptake vary by country, target population and distribution strategy.^11^ The studies available indicate that anxiety about adverse events and the caregiver’s educational level are the greatest emerging barriers to mass drug administration, especially for diseases involving preschool-aged children,^30^^,^^31^ as we found. Consequently, building trust in individuals and the general public plays a critical role in balancing the emotional factors that drive decisions about treatment,^32^ particularly for preventive measures, such as childhood vaccination.^33^ The introduction of targeted awareness initiatives for participants, combined with a strong adverse event management system, could help increase trust and decrease anxiety, thereby boosting the success of mass drug administration programmes. Interestingly, farmers in our study were less willing than non-farmers to participate in repeated preventive chemotherapy, probably because of work constraints, which suggests that the use of integrated health services, for example, rather than short vertical campaigns may increase coverage, as also indicated by studies in Uganda.^34^^,^^35^

Despite structural challenges, collaboration with local health workers, stakeholder partnerships and the efficient use of existing health-care systems enabled us to implement praziquantel preventive chemotherapy in young children with few additional resources.

Scaling up will require a shift in the concept of mass drug administration involving funding and the mutualization of resources and activities across health-care sectors because highly vertical public health strategies remain in place in many low- and middle-income countries.^36^ Currently, the praziquantel that reaches countries where schistosomiasis is endemic is funded by donations and campaigns target mostly school-aged children.^37^ A successful integrated strategy would involve additional costs for investments in optimizing training and monitoring. Our study shows that integrating schistosomiasis activities into existing health structures is feasible. However, cost–effectiveness analyses should be conducted to verify the financial advantages of preventive chemotherapy for national health programmes. Context-specific factors, including cultural and anthropometric differences, must also be considered in generalizing our findings. We found that the venue for praziquantel administration did not seem to play a critical role in the caregiver’s decision to accept treatment, though uptake tended to be lower in hospital settings. This finding should be further investigated to provide guidance on integrating services at the primary care level, especially for vulnerable groups, such as pregnant women.^38^

Our study has several limitations. First, the praziquantel uptake rate was calculated using the number of study participants as the denominator rather than an estimate of the number of children in the same age group in the general population, as suggested by WHO. Consequently, caution is warranted in comparing our findings with programmatic indicators defined by WHO. Second, our use of a convenience, non-probability, sampling strategy could have led to selection bias, which may have affected the external validity and generalizability of our findings. As with any survey, self-reporting is associated with certain biases, such as the influence of the social desirability of the intervention. In addition, recall bias cannot be completely ruled out. Nevertheless, we are confident that the risk was mitigated by the deployment of experienced health workers, training for health workers and continuous supervision. The passive approach adopted for reporting late-onset adverse events may have made monitoring difficult. However, as this was an implementation study, we prioritized integration into the existing national surveillance system for adverse events in an attempt to mimic the real-life scenario of mass drug administration.

To conclude, our study presents a feasible and potentially sustainable model for treating young children with crushed praziquantel tablets. Given that the availability of paediatric praziquantel formulations is often limited, our approach offers a practical alternative for boosting treatment for preschool-aged children. The integration of praziquantel administration into routine paediatric health visits would require few additional resources and would be highly acceptable and tolerable. By helping ensure that every child receives at least one treatment before school age, our strategy would contribute to overall improvements in the health and development of children in settings where schistosomiasis is endemic.
